# Diagnostic Value of Serum Chitinase-3-Like Protein 1 for Liver Fibrosis: A Meta-analysis

**DOI:** 10.1155/2022/3227957

**Published:** 2022-03-20

**Authors:** Xiaoting Huang, Jialing Zhuang, Yongqiang Yang, Jiaxin Jian, Wen Ai, Chunyong Liu, Wenzhi Tang, Changyu Jiang, Yongshen He, Lesheng Huang, Se Peng

**Affiliations:** ^1^Medical Research Center, Huazhong University of Science and Technology Union Shenzhen Hospital, Shenzhen 518051, China; ^2^Department of Laboratory Medicine, Guangdong Provincial Hospital of Chinese Medicine, Zhuhai 519015, China; ^3^Department of Basic Medicine, Zhaoqing Medical College, Zhaoqing 526020, China

## Abstract

**Background:**

Serum chitinase-3-like protein 1 (CHI3L1) is a promising marker for diagnosing liver fibrosis. This meta-analysis was carried out to assess the diagnostic performance of serum CHI3L1 for the estimation of liver fibrosis.

**Methods:**

Systematic searches were performed on PubMed, Embase, Web of Science, Scopus, the Cochrane Library, Google Scholar, Sinomed, the China National Knowledge Infrastructure (CNKI), the Chinese Medical Journal Database, and the Wanfang databases for available studies. The primary studies were screened strictly according to inclusion and exclusion criteria, and sensitivity, specificity, and other measures of accuracy of serum CHI3L1 for evaluating liver fibrosis were pooled with 95% confidence intervals. *I*^2^ was calculated to assess heterogeneity, and sources of heterogeneity were explored by subgroup analysis. Deeks' test was used to assess for publication bias, and likelihood ratio was used to determine posttest probability.

**Results:**

Our research integrated 11 articles, accounting for 1897 patients older than 18 years old. The pooled sensitivity and specificity for significant fibrosis, advanced fibrosis, and cirrhosis were 0.79 and 0.82 with an area under the receiver operating characteristic curve (AUC) of 0.85, 0.81 and 0.83 with an AUC of 0.91, and 0.72 and 0.74 with an AUC of 0.85, respectively. Random-effects models were used to assess for significant heterogeneity, and subgroup analysis showed that age and aetiology of included patients were likely sources of heterogeneity. No potential publication bias was found for serum CHI3L1 in the diagnosis of significant fibrosis, advanced fibrosis, or cirrhosis, and posttest probability was moderate.

**Conclusion:**

Measurement of serum CHI3L1 is a feasible diagnostic tool for liver fibrosis.

## 1. Introduction

Liver fibrosis is a severe health problem globally, with significant morbidity and mortality, and its incidence is on the increase in both adults and children [1–5]. Steatohepatitis, drug-induced and immune liver disease, chronic liver infection, schistosomiasis, and alcohol abuse all result in liver fibrosis. Among them, chronic infection with hepatitis virus is the major known risk factor for liver fibrosis [6–11]. Clinically, hepatic fibrosis can process to cirrhosis or liver cancer, which can be life-threatening [12–16]. Fortunately, accumulating evidence suggests that liver fibrosis is reversible if treated in the early stage. However, there is no effective and specific medicine for the treatment of liver fibrosis in clinical practice; therefore, timely and comprehensive diagnosis has been critical to reduce its progression.

As the gold standard of liver fibrosis diagnosis, liver biopsy is a markedly matured testing technology. However, its high costs, invasive examination, risk of complications, and sampling error restrict its clinical application [15, 17–19]. Transient elastography (TE) has excellent diagnostic accuracy for liver fibrosis, but TE is proven to be accurate for the diagnosis of severe fibrosis and cirrhosis only [20–22]. Other noninvasive markers have been applied widely to evaluate fibrosis, but many of them are found to be nonspecific. Therefore, finding a clinical diagnostic indicator that is noninvasive, safe, inexpensive, specific, and convenient has crucial clinical significance for diagnosing and monitoring liver fibrosis.

Serum chitinase-3-like protein 1 (CHI3L1, YKL-40 protein, breast regression protein-39, or human cartilage glycoprotein-39) is a member of the mammalian chitinase family that is highly liver-specific and involved in inflammation, cell proliferation, and differentiation [23–26]. Studies have indicated that serum CHI3L1 level in patients with liver fibrosis is significantly higher than in healthy controls, and that high levels of CHI3L1 correlate with the severity of fibrosis [23, 25], suggesting that CHI3L1 plays an important role in liver fibrosis. The clinical diagnosis of liver fibrosis has been found that the sensitivity of CHI3L1 as an indicator of liver fibrosis was 27% higher than indicators type III procollagen, type IV collagen, laminin, and hyaluronidase and 22% higher than FibroScan [25]. In addition, serum CHI3L1 has good correlation with TE and better consistency with liver pathology [20, 21]. Serum CHI3L1 has already been recommended at international professional conferences as a noninvasive diagnostic technology for liver fibrosis and has been included in the “Guidelines on the Management of Hepatic Encephalopathy in Cirrhosis” of the Chinese Medical Association (2018) and the “Guidelines on the Prevention and Treatment in Chronic Hepatitis B” of the Chinese Medical Association (2019) [27, 28].

In recent years, researchers have conducted meta-analyses of the diagnostic value of serum CHI3L1 for assessing various diseases, including coronary artery disease, rectal cancer, psoriasis, and diabetic nephropathy in patients with type 2 diabetes mellitus [24, 29–35]. In addition, two published meta-analyses have investigated the use of serum CHI3L1 combined with hyaluronic acid and FIBROSpect II (FS-II) for discriminating fibrosis stages in chronic hepatitis C [36, 37]. An article published in 2018 reported a meta-analytic study of the diagnostic value of YKL-40 (CHI3L1) for liver fibrosis, with the same objective as the present study. However, their findings showed that the diagnostic value of serum CHI3L1 for significant and advanced fibrosis was limited by low sensitivity and specificity. In addition, analyses of heterogeneity, publication bias, and posttest probability were lacking [38]. In this regard, it is worth conducting an updated and more comprehensive meta-analysis to identify the value of serum CHI3L1 level in the assessment of liver fibrosis.

## 2. Methods

The protocol for this meta-analysis was registered on PROSPERO (CRD42021247959). We followed reporting guidance from the Preferred Reporting Items for Systematic Reviews and Meta-Analyses (PRISMA) statement for this study [39, 40].

### 2.1. Search Strategy

A systematic search was conducted in PubMed, Embase, Web of Science, Scopus, Cochrane Library, Google Scholar, Sinomed, the China National Knowledge Infrastructure (CNKI), the Chinese Medical Journal Database, and the Wanfang databases for relevant articles published between Jan 1, 2000, and Oct 31, 2021. In addition, we searched grey literature by reviewing the references of primary studies and related review articles from major conferences manually. We used the medical subject headings (MeSH) terms in PubMed using the search keywords “Chitinase-3-like protein 1”, “CHI3L1”, “YLK-40 protein”, “Cartilage Glycoprotein 39”, “GP-39 Protein”, “hepatic fibrosis”, “liver cirrhosis”, “liver fibrosis”, “diagnosis”, “sensitivity and specificity”, “predictive value of tests”, and “accuracy” combined together using OR and/or AND for a standard search. The strategy was modified according to each specific database, including Embase, Web of Science, Scopus, the Cochrane Library, Google Scholar, and Sinomed, to obtain the most relevant results. For the CNKI, the Chinese Medical Journal Database, and the Wanfang databases, the key words were combined with free words for searching. The titles and abstracts were read independently by two investigators (XT Huang and JL Zhuang), who selected literature for a second round of screening. The second screening was based on the full texts, to determine whether the retrieved articles were suitable for inclusion in the study. The detailed search strategies are showed in Supplementary Table 1.

### 2.2. Inclusion and Exclusion Criteria

**Figure 1:**
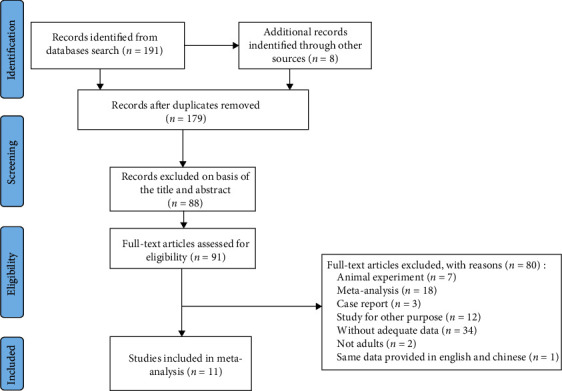
PRISMA flow chart of the study selection process for eligible studies.

There were no restrictions on language in the selection and determination of original research. The articles included in this study had the following characteristics: (1) study patients were adults (over 18 years); (2) patients with liver fibrosis from various causes were accepted (e.g., hepatitis B virus (HBV), hepatitis C virus (HCV), nonalcoholic fatty liver disease, autoimmune hepatitis, and any other cause); (3) histopathologic analysis was used as the reference standard; (4) studies provided sufficient information to calculate the sensitivity and specificity data on CHI3L1 directly and indirectly. The exclusion criteria were as follows: (1) any article that was a duplication, animal experiment, single case report, meta-analysis, or review article; (2) articles without adequate data; (3) articles lacking pathological gold standard confirmation results. The flow chart of study selection is shown in Figure 1.

### 2.3. Definition of Liver Fibrosis

A fibrosis scoring system was devised that was similar to the METAVIR scoring system and included five stages: stage 0 = no fibrosis, stage 1 = fibrosis in any localization but without septa, stage 2 = few septa, stage 3 = many septa, and stage 4 = cirrhosis [41]. Commonly, significant fibrosis, advanced fibrosis, and cirrhosis are defined as stages F2–F4, F3–F4, and F4 by METAVIR and the Batts–Ludwig scoring system; S2–S4, S3–S4, and S4 by the Scheuer scoring system; and staged as F3–F6, F4–F6, and F5–F6 by the Ishak scoring system, respectively [11, 42–45]. With similar histological definitions for liver fibrosis in our study, significant fibrosis was accepted as a ≥F2 METAVIR score, a ≥ S2 Scheuer score, or a ≥ F3 Ishak score; advanced fibrosis as a ≥ F3 METAVIR score, a ≥ S3 Scheuer score, or a ≥ F4 Ishak score; cirrhosis as a METAVIR score of F4, an F4 Scheuer score, or an Ishak score of F6.

### 2.4. Data Extraction and Quality Assessment

All included studies were extracted and summarized by XT Huang and JL Zhuang independently. Each investigator recorded the sensitivity, specificity, positive predictive value (PPV), negative predictive value (NPV), and receiver operating characteristic (ROC) curve values from the included studies. Data on author, publication year, sample size for different stages of liver fibrosis, assay method, gold standard, average age, sex, aetiology, and cut-off points for serum CHI3L1 from each report were also extracted. Any discrepancies were resolved by referral to a third investigator (CY Liu).

We evaluated the quality of the 11 articles that were finally selected using the QUADAS-2 tool, which has four domains: patient selection, index test, reference standard, and flow and timing. We tailored the guidelines for scoring each item on the checklist to our review. Finally, all items were categorized as low, unclear, or high risk of bias.

### 2.5. Data Analysis

We evaluated the quality of the selected studies using Review Manager (version 5.2). We used Meta-Disc (version 1.4.0) and STATA (version 14.0) to conduct the statistical analyses. *P* values < 0.05 were considered statistically significant.

We tabulated true-positive, false-negative, false-positive, and true-negative from included studies. The summary of diagnostic accuracy was computed for each study: sensitivity (true-positive), specificity (1-false-positive), positive likelihood ratio (PLR), negative likelihood ratio (NLR), diagnostic odds ratio (DOR), and the area under the ROC (AUROC) curve, with 95% confidence interval (CI) calculated for each analysis.

### 2.6. Assessment of Heterogeneity and Publication Bias

Spearman's correlation coefficient was calculated to evaluate the threshold effect firstly. With threshold effect excluded, the heterogeneity between studies was assessed with an inconsistency index (*I*^2^ statistic). *I*^2^ greater than 50% was selected as a marker for substantial heterogeneity, for which the random-effects model can be chosen and employed. Potential sources of heterogeneity were explored by subgroup analyses. Subgroup analysis for stage F1 was not performed because the F1 sample size was too small. Hence, only the results of stages F2–F4 are shown. Subgroup analyses were performed according to mean age (under 40 years vs. over 40 years), aetiology (HBV vs. other), and scoring system (METAVIR vs. other).

For evaluation of publication bias, we employed a linear regression analysis of funnel plot asymmetry, using a Deeks' plot. Finally, we used pooled likelihood ratios to determine the posttest probability, using STATA 14.0.

## 3. Results

### 3.1. Search Results

Our initial literature search identified 191 publications through the comprehensive electronic search, and eight additional (grey) literature items were screened manually from the references of the included studies and other articles from major conferences. We first removed 19 duplicate studies, 88 articles were excluded after reviewing the titles and abstracts of all the articles, and then, 80 articles were excluded by reviewing the full texts carefully. Eventually, 11 studies with a total of 1897 patients were considered eligible for inclusion in the analysis. The 11 studies had been performed in the United States, France, Japan, and China. The main features of each included study are presented in Table 1 and Supplementary Table 2. Supplementary Table 2 provides the detailed characteristics such as cut-off and AUROC of all the stages of the liver fibrosis for each included studies.

### 3.2. Characteristics of Each Stage of Liver Fibrosis

Two articles reported on diagnosing mild fibrosis (*F* ≥ 1) using serum CHI3L1 with a total of 519 patients, with mean ages greater than 40 years. Six articles reported on diagnosing significant fibrosis (*F* ≥ 2) using serum CHI3L1 with a total of 1193 patients, with mean ages between 24.5 and 52.0 years. Diagnosing advanced fibrosis (*F* ≥ 3) by serum CHI3L1 level was described in four papers, with a total of 1020 patients of mean ages between 24.5 and 65.8 years. Six articles reported on diagnosing cirrhosis (*F* = 4) using serum CHI3L1, with a total of 907 patients of mean ages from 39.0 to 52.2 years.

### 3.3. Quality Evaluation

**Figure 2:**
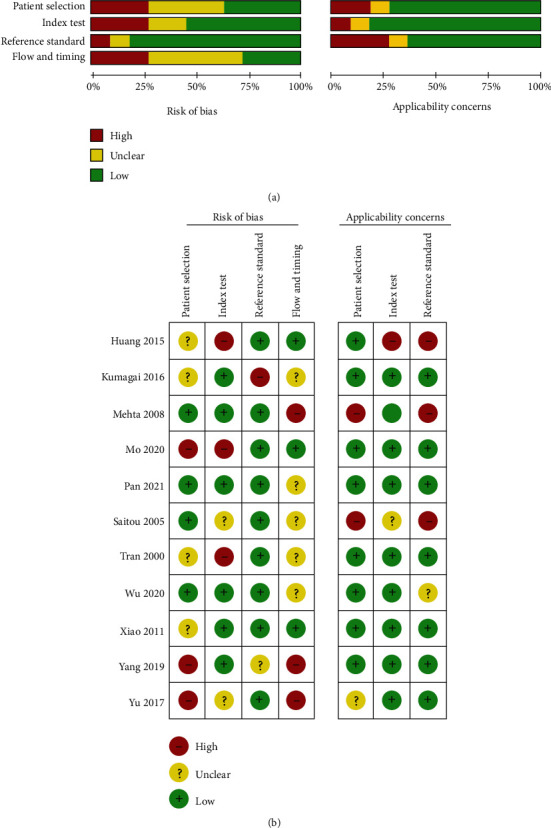
Quality assessment of the individual studies: (a) overall quality assessment of the included studies; (b) quality assessment of the individual studies.

To minimize the risk of bias, the QUADAS-2 tool was used to assess the quality of the included studies. This tool comprises four domains; each domain is assessed in terms of risk of bias, and the first three domains are also assessed in terms of concerns about applicability [56–59]. Overall, no study fulfilled all the criteria, many of them were assessed as of high quality (Figures 2(a) and 2(b)). Specifically, eight studies (73%) met at least 50% of all the items [26, 47–49, 51–54], and two studies met six items (86%) especially [49, 53]. However, the remaining three studies were considered of low quality, as whether enrolled patients were sampled consecutively was unclear, and the included patients, conduct of the study, and interpretation did not match the review questions exactly [46, 50, 55].

### 3.4. Pooled Diagnostic Accuracy

The summary ROC (SROC) curve can be considered as the average value for sensitivity for a test over all possible specificity values, presenting a global summary of test performance [22, 59]. Usually, at least three records are needed to estimate the summary AUROC curve values, and a forest plot cannot be established unless there are more than four sets of data. In consideration of the established statistical regulations, our study analyzed the diagnostic values of fibrosis stages with significant fibrosis (*F* ≥ 2), advanced fibrosis (*F* ≥ 3), and cirrhosis (F4).

A random-effects model was used to determine the diagnostic accuracy of serum CHI3L1 for diagnosing stages of liver fibrosis, because heterogeneity (*I*^2^ values for sensitivity, specificity, PLR, NLR, and DOR) for fibrosis stages F2, F3, and F4 was greater than 50%. The main results for each stage follow.

#### 3.4.1. Detecting Significant Fibrosis (*F* ≥ 2)

The pooled sensitivity and specificity for significant fibrosis (*F* ≥ 2) were 0.79 (95% CI = 0.75 to 0.82) and 0.82 (95% CI = 0.78 to 0.85) with an AUC of 0.85; the PLR and NLR were 3.65 (95% CI = 2.00 to 6.66) and 0.29 (95% CI = 0.19 to 0.44), and the DOR was 12.98 (95% CI = 5.75 to 29.32, *I*^2^ = 84.4%).

#### 3.4.2. Detecting Advanced Fibrosis (*F* ≥ 3)

The pooled sensitivity and specificity for advanced fibrosis (*F* ≥ 3) were 0.81 (95% CI = 0.77 to 0.84) and 0.83 (95% CI = 0.80 to 0.86) with an AUC of 0.91; the PLR and NLR were 5.25 (95% CI = 2.97 to 9.37) and 0.19 (95% CI = 0.09 to 0.41), and the DOR was 28.38 (95% CI = 7.33 to 109.91, *I*^2^ = 90.9%).

#### 3.4.3. Detecting Cirrhosis (*F* = 4)

**Figure 3:**
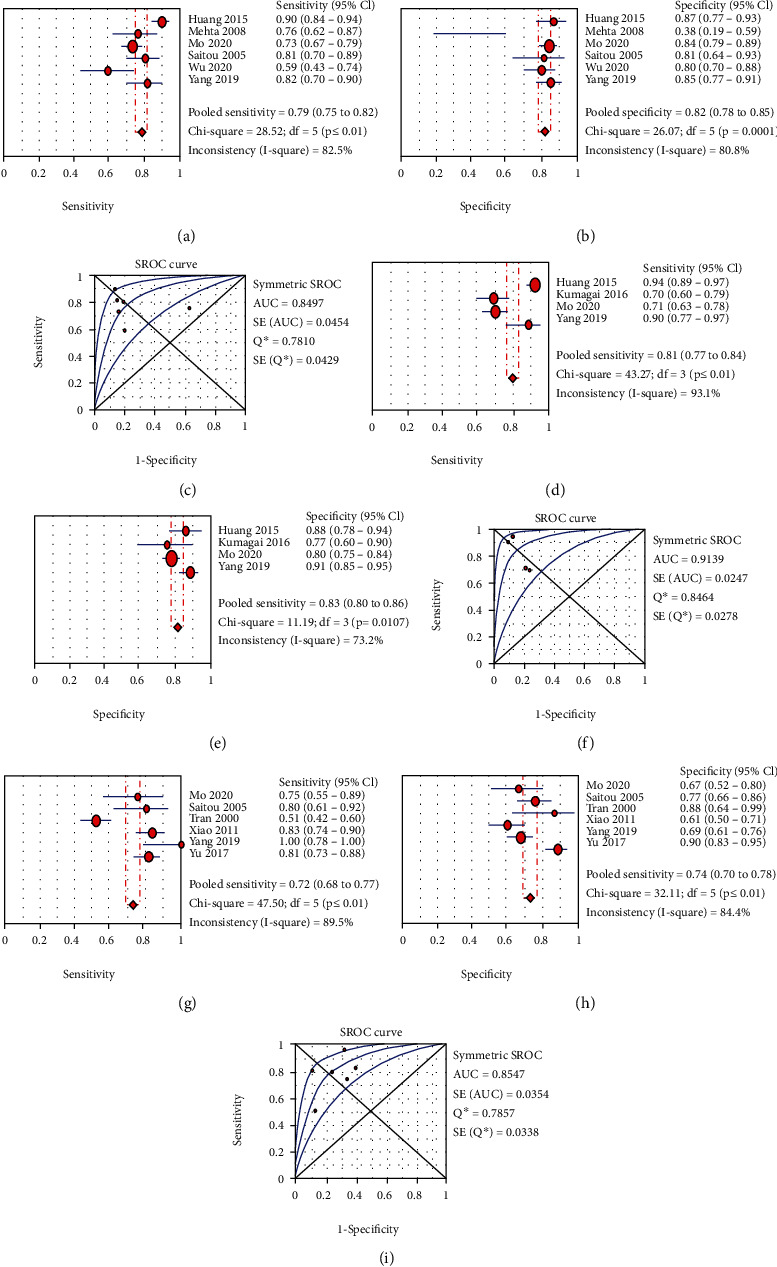
(a, b) Diagnostic accuracy of CHI3L1 for significant fibrosis (*F* ≥ 2); (c) SROC curve of CHI3L1 for detecting significant fibrosis; (d, e) diagnostic accuracy of CHI3L1 for advanced fibrosis (*F* ≥ 3); (f) SROC curve of CHI3L1 for detecting advanced fibrosis; (g, h) diagnostic accuracy of CHI3L1 for cirrhosis (F4); (i) SROC curve of CHI3L1 for detecting cirrhosis.

The pooled sensitivity and specificity for cirrhosis (*F* = 4) were 0.72 (95% CI = 0.68 to 0.77) and 0.74 (95% CI = 0.70 to 0.78) with an AUC of 0.85; the PLR and NLR were 3.32 (95% CI = 2.27 to 4.85) and 0.29 (95% CI = 0.16 to 0.53), and the DOR was 13.41 (95% CI = 6.40 to 28.11, *I*^2^ = 63.70%). Figures 3(a)–3(i) show the pooled sensitivity, specificity, and SROC curve of three stages of fibrosis in liver fibrosis patients.

### 3.5. Heterogeneity Analysis and Subgroup Analysis

In our study, the Spearman's correlation coefficients were –0.714, –0.80, and 0.314 for stages F2, F3, and F4, respectively, with *P* values greater than 0.05, suggesting that the threshold effect caused no heterogeneity. Heterogeneity was evaluated by the *I*^2^ statistic, with *I*^2^ values exceeding 25%, 50%, and 75% representing low, moderate, and high heterogeneity, respectively [60, 61]. This meta-analysis, the *I*^2^ values for sensitivity, specificity, PLR, NLR, and DOR for fibrosis stages F2, F3, and F4 were over 50%, suggesting that high heterogeneity was presented.

In view of the significant heterogeneity, we conducted a subgroup analysis to investigate the potential sources of heterogeneity by age, aetiology, and scoring system factors. This was not conducted for stage F2 because only two studies included this stage. The detailed subgroup analysis results are listed in Table 2. As presented in Table 2, for stages F2, F3, and F4, the results of mean age group are same as the aetiology group. For instance, in the subgroup of advanced fibrosis (*F* ≥ 3), two studies that included patients under 40 years showed a pooled sensitivity and specificity of 0.93 (0.89–0.96) and 0.90 (0.85–0.94) in identifying advanced liver fibrosis in patients with HBV. In addition, two studies that utilized a non-METAVIR scoring system had a pooled sensitivity of 0.83 (0.78–0.87) and specificity of 0.81 (0.77–0.85), which was better than the METAVIR scoring system. Moreover, in the subgroups of mean age and aetiology, the heterogeneity between studies was very low (*I*^2^ = 0%), but this did not appear in the scoring system group.

In this regard, for all the stages of fibrosis, age and aetiology could be considered as sources of heterogeneity, which influenced the reported diagnostic accuracy of the test.

### 3.6. Publication Bias

**Figure 4:**
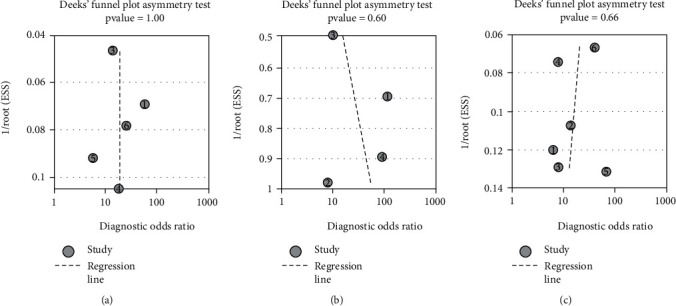
Linear regression test of funnel plot asymmetry for publications: (a) significant fibrosis (*P* = 1.00); (b) advanced fibrosis (*P* = 0.60); (c) cirrhosis (*P* = 0.66).

A linear regression test of funnel plot asymmetry can be performed with more than two sets of data, and if publication bias is present, the plot can appear asymmetric [10, 62]. We identified publication bias by using Deeks' funnel plot asymmetry regression test with STATA, with *P* value of <0.05 considered the criterion for statistical significance. Here, Deeks' regression test showed no asymmetrical distribution in the funnel plots, indicating insufficient evidence of publication bias for articles on use of serum CHI3L1 in diagnosing significant fibrosis, advanced fibrosis, and cirrhosis, with *P* values of 1.00, 0.60, and 0.66, respectively (Figures 4(a)–4(c)).

### 3.7. Post-test Probability

**Figure 5:**
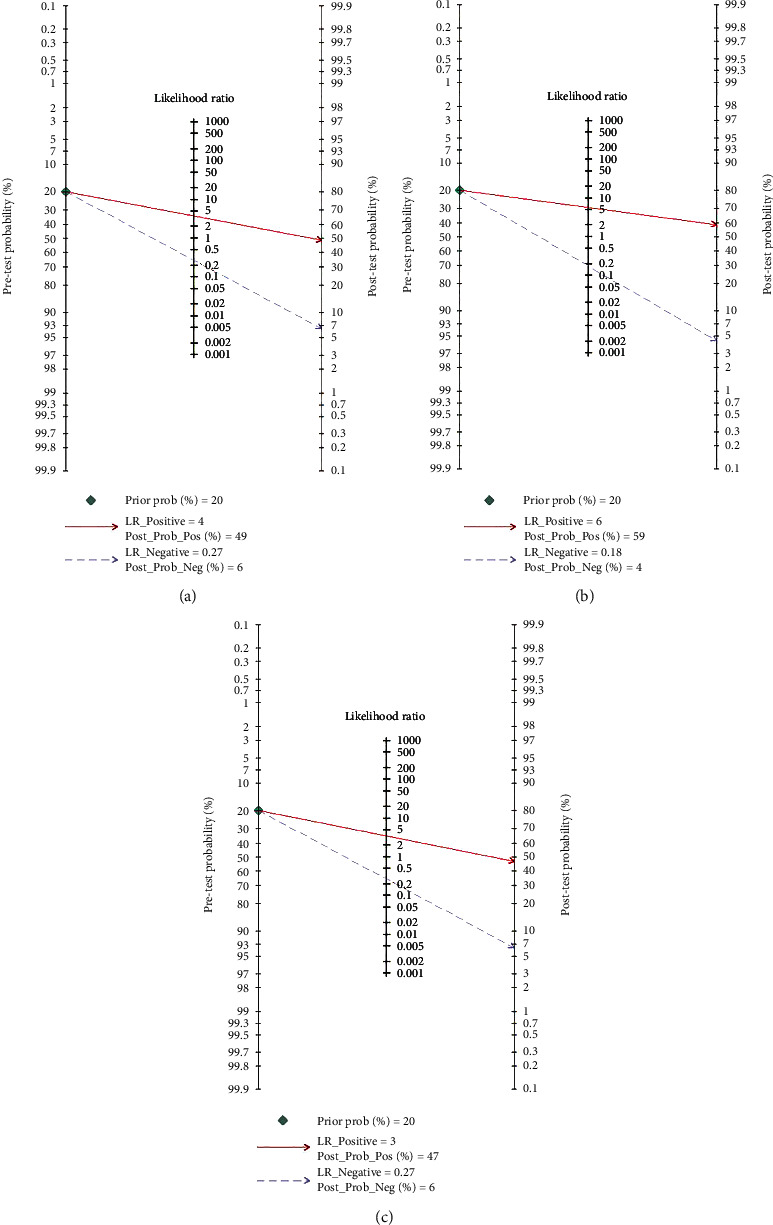
Fagan nomogram of serum CHI3L1 for diagnosis of liver fibrosis: (a) significant fibrosis; (b) advanced fibrosis; (c) cirrhosis.

Post-test probability is relevant to clinicians, and we therefore used likelihood ratios to determine posttest probability for the positive and negative index test results [63, 64]. In our study, a Fagan nomogram (Figures 5(a)–5(c)) revealed a prior probability of 20% and posttest probability of 49% when the result of serum CHI3L1 was positive for a significant fibrosis diagnosis and 6% when the results were negative. Similarly, the posttest probabilities were 59% and 47% when serum CHI3L1 was positive for advanced fibrosis and cirrhosis diagnosis, respectively, and 4% and 6% when the results were negative for advanced fibrosis and cirrhosis diagnosis.

## 4. Discussion

An early diagnosis is critical in liver fibrosis. In this meta-analysis, we addressed the diagnostic value of serum CHI3L1 as a promising and strong noninvasive marker of predicting hepatic fibrosis.

Until now, a series of clinical diagnostic techniques such as liver biopsy, B-mode ultrasonography, TE, and conventional serum markers (type III procollagen, type IV collagen, laminin, and hyaluronidase) have been devised for liver fibrosis diagnosis and assessment [65, 66]. However, these methods have disadvantages that include invasiveness, high cost burden, and a lack of specificity. Therefore, in accordance with modern concepts of safety, affordability, and convenience, finding a satisfactory screening method is necessary. Serum CHI3L1 has been identified as a potential marker of liver fibrosis in recent years, with several studies demonstrating that CHI3L1 serves as the upstream signalling molecule regulating liver fibrosis [67]. This indicates the potential of using CHI3L1 to make an early diagnosis of liver fibrosis.

A prior meta-analysis that included nine articles published before 2018 evaluated the diagnostic value of serum YKL-40 (CHI3L1) for liver fibrosis stage. However, Zhang and colleagues [68] concluded that its diagnostic value for significant and advanced fibrosis was limited. They included patients undergoing kidney transplant in their study, and the specificity for significant fibrosis was as low as 0.53, which was poorer than the results of specificity in our study [69]. Actually, serum CHI3L1 concentration is closely related to kidney function, so including kidney transplant patients included in their analysis will lead to biased findings. In addition, they did not perform publication bias, sensitivity, and posttest analyses. In this study, we performed an updated and more comprehensive meta-analysis to get a better view of the diagnostic value of CHI3L1 in liver fibrosis.

To avoid selection bias in our study, we conducted a thorough literature review of the performance of CHI3L1 in liver fibrosis diagnosis in worldwide databases. A manual search of the African Index Medicus (MIX, https://iifphc.org/acadp_listings/african-index-medicus/) was also conducted but failed to obtain relevant studies. Related references of the initially screened articles and previous systematic reviews were searched manually for grey literature. As a result, 11 studies were reserved for our meta-analysis. By reading the full texts of the included studies, we were able to identify how the different stages of liver fibrosis were reported using the Scheur, METAVIR, Ishak, and HAI systems. Since the histological definitions of liver fibrosis were similar [7, 70–73], we unified the stages of liver fibrosis to mild fibrosis (*F* ≥ 1), significant fibrosis (*F* ≥ 2), advanced fibrosis (*F* ≥ 3), and cirrhosis (F4). However, only two studies discussed mild fibrosis (*F* ≥ 1), which made pooled analysis in this group impossible. As these two studies fulfilled the inclusion criteria, we listed their main data in Table 1 and evaluated their quality. The QUADAS-2 tool was used to evaluate the quality of all the included studies. On the basis of our strict inclusion criteria, many studies were of very high quality, with 9 papers of the 11 meeting at least 50% of the items.

Sensitivity shows the ability of a test to correctly identify those with the infection or disease, and specificity is the opposite. The SROC curve can be considered as the average sensitivity of a test over all possible values of specificity, presenting a synthetic summary of test performance [74, 75]. In the prediction of serum CHI3L1 for liver fibrosis in this study, we found that the summary sensitivity and specificity of advanced fibrosis (*F* ≥ 3) were higher than the significant fibrosis (*F* ≥ 2) and cirrhosis (F4). Interestingly, the AUC value for advanced fibrosis was greater than 0.90, validating that the serum CHI3L1 has the greatest diagnostic ability to predict the advanced fibrosis in clinical. In addition, the pooled diagnostic values were significantly greater than those for the indicators APRI index and FIB-4 index reported by Jiang et al. [68]. The findings of PLR and NLR for all stages show the great discriminant ability in liver fibrosis diagnosis. Correspondingly, we observed the good performance of CHI3L1 in diagnosing liver fibrosis through the ideal results of pooled DOR values in our work. Overall, there was a positive correlation between serum CHI3L1 and hepatic fibrosis. The results in our study showed that when patients with advanced fibrosis (F3) were compared to those with significant fibrosis (F2) and cirrhosis (F4), serum CHI3L1 had the highest diagnostic accuracy for F3 at an early age. All the results imply that serum CHI3L1 offered the best diagnostic performance in the diagnosis of advanced fibrosis. These observations are similar to those of Das et al., Rath et al., and Zhou et al. [6, 71, 72].

Threshold effect is commonly explored by calculating Spearman's correlation coefficient. No threshold effect was found in our study. However, results from the *I*^2^ test indicated significant heterogeneity (*I*^2^ was greater than 50% for stages F1, F2, and F3), meaning that a random-effects model was selected for the analysis. A meta-regression analysis is an ideal way to identify the heterogeneity sources where heterogeneity is high; however, groups of more than 10 studies with complete data are required [59]. The included studies for each stage for liver fibrosis in this study were less than 10, so we instead chose subgroup analysis to find the underlying causes of the heterogeneity. According to the calculation, results were similar for mean age group and aetiology group. We approached this interesting finding by searching for papers on the relationship between HBV infection and age. Collective evidence suggested that age is a key factor in determining the risk of chronic infection such as chronic hepatitis B (CHB), with older persons especially at risk for progressive disease such as liver fibrosis [69]. Yan and colleagues [9] showed that liver fibrosis occurs in more than 30% of CHB patients. These studies suggest that CHB is closely related to liver fibrosis, and liver disease aetiology is associated with age. Therefore, we guessed that aetiology and age may account for the sources of heterogeneity.

In our study, bias in the publication of literature was not detected, with *P* values for significant fibrosis, advanced fibrosis, and cirrhosis greater than 0.05. In addition, the symmetrical distribution of the funnel plots with the *P* values suggested that the heterogeneity did not result from publication bias [77].

Posttest probability and likelihood ratio were used to assess the accuracy of diagnosis prevalence for the method we tested, for patients with a positive or negative test. In our report, the likelihood ratio and posttest probabilities were moderate for significant fibrosis, advanced fibrosis, and cirrhosis.

Our study had certain limitations. First, the sample sizes in some of the studies were small. We considered papers with patient samples of less than 100 carefully and still included them if they strictly fulfilled the inclusion criteria. Second, meta-regression analysis to explore heterogeneity was not possible because of the limited number of articles, so we conducted a subgroup analysis instead. Third, most of the included articles were from Europe and Asia, especially China, which may limit the generalizability of our results. However, although liver disease has become a significant health problem worldwide, China has the world's largest burden of liver disease, with statistics for 2017 showing that the absolute number of HBV-infected people in China is around 70 million. Most of included articles in our study were reports of studies performed in China, which is consistent with the geographical distribution of the disease [1, 10, 76, 77]. This observation underlines also the lack of related data from other regions, such as North America and Africa. Finally, we acknowledge that the topic in this paper has some limitations on novelty and innovation. Many studies have been exploring the diagnostic value of serum CHI3L1 for liver fibrosis, but we noticed that the diagnostic value of CHI3L1 is controversial. Our report showed that the CHI3L1 is a feasible indicator for liver fibrosis diagnosis, especially for the advanced fibrosis. The result is different from the prior meta-analysis, which showing the diagnostic value of CHI3L1 for significant and advanced fibrosis is limited. More importantly, this study is of great significance in clinical application. High-quality studies with larger sample sizes are required on the diagnostic value of CHI3L1 in liver fibrosis, with multiregional cooperation.

Despite these limitations, our study provides evidence on the particular advantages of serum CHI3L1 over other indicators [69, 70, 72, 73], and the association between serum CHI3L1 and liver fibrosis diagnosis was a worthy topic for meta-analysis.

Serum CHI3L1 appears to be an excellent indicator in the diagnosis of the liver fibrosis. Of course, measurement of serum CHI3L1 cannot completely replace liver biopsy, and some patients will still need invasive examination to confirm diagnosis, making the continued development of noninvasive diagnostic methods for liver fibrosis even more necessary. To guide clinical interventions accurately, further high-quality studies on the diagnosis of liver fibrosis, with larger sample sizes, are needed.

## 5. Conclusions

In summary, measurement of serum CHI3L1 is emerging as a powerful tool for detecting liver fibrosis, especially in advanced fibrosis. Use of this biomarker has many advantages, including noninvasiveness, expeditiousness, and accuracy. Following recommendations from international professional conferences, wide use of serum CHI3L as a marker of liver fibrosis is expected. However, given the limitations mentioned in our meta-analysis, serum CHI3L1 cannot completely replace the “gold standard” of liver biopsy in the diagnosis of liver fibrosis, and further larger-scale research with multiregional cooperation is needed to confirm the practicality and validity of serum CHI3L1 as a liver fibrosis marker.

## Figures and Tables

**Table 1:** Characteristics of the included studies (*n* = 11).

First author	Year	Country	Reference method	Test method	Male, *n* (%)	Average age, y	Scoring system	Aetiology
Huang[46]	2015	China	Liver biopsy	ELISA	NA	24.5	Scheuer	HBV
Kumagai [47]	2016	Japan	Liver biopsy	ELISA	59 (44.0)	65.8	METAVIR	Mixed
Mehta[26]	2008	America	Liver biopsy	ELISA	46 (62.0)	52.0	Ishak	HCV
Mo [48]	2020	China	Liver biopsy	ELISA	257 (55.8)	40.9	Scheuer	Mixed
Pan [49]	2021	China	Liver biopsy	Fluorescence immunochromatography	26 (76.5)	48.5	Scheuer	Mixed
Saitou[50]	2005	Japan	Liver biopsy	ELISA	62 (56.9)	51.4	METAVIR	HCV
Tran [51]	2000	France	Liver biopsy	ELISA	106 (72.6)	49.2	NA	Mixed
Wu [52]	2020	China	Liver biopsy	ELISA	90 (67.2)	39.0	METAVIR	HBV
Xiao [53]	2011	China	Liver biopsy	ELISA	80 (44.4)	39.8	HAI	HBV
Yang [54]	2019	China	Liver biopsy	ELISA	86 (50.0)	39.0	METAVIR	HBV
Yu [55]	2017	China	Liver biopsy	ELISA	172 (76.1)	52.2	NA	Mixed

ELISA: enzyme-linked immunoassay; HBV: hepatitis B virus; HCV: hepatitis C virus; NA: not available; HAI: histological activity index.

**Table 2:** Subgroup analysis of serum CHI3L1 in diagnosing stages of liver fibrosis.

Subgroup	Significant fibrosis (*F* ≥ 2)			Advanced fibrosis (*F* ≥ 3)			Cirrhosis (*F* = 4)		
	Sensitivity (95% CI)	Specificity (95% CI)	AUC	Sensitivity (95% CI)	Specificity (95% CI)	AUC	Sensitivity (95% CI)	Specificity (95% CI)	AUC
*I* ^2^	82.5%	80.8%	—	93.1%	73.5%	—	89.5%	84.4%	—
Age									
Under 40	0.83 (0.78-0.87)	0.84 (0.79-0.88)	0.92	0.93 (0.89-0.96)	0.90 (0.85-0.94)	0.50	0.86 (0.78-0.92)	0.66 (0.60-0.72)	0.85
*I* ^2^	90.3%	0%	—	0%	0%	—	80.1%	32.9%	—
Over 40	0.75 (0.70-0.79)	0.80 (0.75-0.84)	0.82	0.70 (0.64-0.83)	0.79 (0.75-0.84)	0.50	0.68 (0.62-0.73)	0.82 (0.77-0.87)	0.50
*I* ^2^	0%	91.3%	—	0%	0%	—	89.5%	77.9%	—
Aetiology									
HBV	0.83 (0.78-0.87)	0.84 (0.79-0.88)	0.92	0.93 (0.89-0.96)	0.90 (0.85-0.94)	0.50	0.86 (0.78-0.92)	0.66 (0.60-0.72)	0.85
*I* ^2^	90.3%	0	—	0%	0%	—	80.1%	32.9%	—
Other	0.75 (0.70-0.79)	0.80 (0.75-0.84)	0.82	0.70 (0.64-0.83)	0.79 (0.75-0.84)	0.50	0.68 (0.62-0.73)	0.82 (0.77-0.87)	0.50
*I* ^2^	0	91.3%	—	0%	0%	—	89.5%	77.9%	—
Scoring system									
METAVIR	0.80 (0.76-0.83)	0.81 (0.77-0.85)	0.88	0.76 (0.68-0.83)	0.88 (0.82-0.92)	0.50	0.87 (0.73-0.95)	0.72 (0.65-0.77)	0.83
*I* ^2^	89.6%	92.0%	—	86.8%	76.6%	—	81.2%	46.8%	—
Other	0.76 (0.69-0.82)	0.83 (0.77-0.87)	0.91	0.83 (0.78-0.87)	0.81 (0.77-0.85)	0.50	0.70 (0.65-0.75)	0.76 (0.71-0.81)	0.50
*I* ^2^	75.5%	0%	—	96.9%	66.7%	—	91.7%	89.6%	—

## Data Availability

No data were used to support this study.

## Supplementary Materials

Supplementary 1 Supplementary Table 1: search strategy for all databases.

Supplementary 2 Supplementary Table 2: characteristics of the included studies.
