# Supplementary material for: Diagnostic Value of Serum Chitinase-3-Like Protein 1 for Liver Fibrosis: A Meta-analysis
Source: Biomed Res Int. 2022 Mar 20;2022:3227957. doi: 10.1155/2022/3227957 (PMC8961437; doi:10.1155/2022/3227957)
Supplement: Supplementary 1 — Supplementary Table 1: search strategy for all databases. [file 3227957.f1.docx]

Supplementary Table 1. Search strategy for all databases

| ***Search strategy for PubMed***  (((("Chitinase-3-Like Protein 1"[Mesh]) OR ((Chitinase 3 Like Protein 1[Title/Abstract]) OR (Cartilage Glycoprotein 39[Title/Abstract]) OR (Glycoprotein 39, Cartilage[Title/Abstract]) OR (GP-39 Protein[Title/Abstract]) OR (GP 39 Protein[Title/Abstract]) OR (YLK-40 Protein[Title/Abstract]) OR (YLK 40 Protein[Title/Abstract]) OR (CHI3L1 Protein[Title/Abstract]) OR (CGP-39 Protein[Title/Abstract]) OR (CGP 39 Protein[Title/Abstract]))) AND ("Liver Cirrhosis"[Mesh])) OR ((Hepatic Cirrhosis[Title/Abstract]) OR (Cirrhosis, Hepatic[Title/Abstract]) OR (Cirrhosis, Liver[Title/Abstract]) OR (Fibrosis, Liver[Title/Abstract]) OR (Liver Fibrosis[Title/Abstract]))) AND (sensitiv*[Title/Abstract] OR sensitivity and specificity [MeSH Terms] OR (predictive[Title/Abstract] AND value*[Title/Abstract]) OR predictive value of tests[MeSH Term] OR accuracy*[Title/Abstract]). |
| --- |
| ***Search strategy for Embase***  (1)'Hepatic Cirrhosis':ab,ti OR 'Cirrhosis, Hepatic':ab,ti OR 'Cirrhosis, Liver':ab,ti OR 'Fibrosis, Liver':ab,ti OR 'Liver Fibrosis':ab,ti  (2)'Chitinase 3 Like Protein 1':ab,ti OR 'Cartilage Glycoprotein 39':ab,ti OR 'Glycoprotein 39, Cartilage':ab,ti OR 'GP-39 Protein':ab,ti OR 'GP 39 Protein':ab,ti OR 'YLK-40 Protein':ab,ti OR 'YLK 40 Protein':ab,ti OR 'CHI3L1 Protein':ab,ti OR 'CGP-39 Protein':ab,ti OR 'CGP 39 Protein':ab,ti  (3)'sensitiv':ab,ti OR 'sensitivity and specificity':ab,ti OR 'predictive':ab,ti OR 'predictive value of tests':ab,ti OR 'accuracy':ab,ti |
| ***Search strategy for Web of Science***  (1)(Liver Cirrhosis or Hepatic Cirrhosis or Cirrhosis, Hepatic or Cirrhosis, Liver or Fibrosis, Liver or Liver Fibrosis)  (2)(Chitinase 3-like protein 1 or Chitinase 3 Like Protein 1 or Cartilage Glycoprotein 39 or Glycoprotein 39, Cartilage or GP-39 Protein or GP 39 Protein or YLK-40 Protein or YLK 40 Protein or CHI3L1 Protein or CGP-39 Protein or CGP 39 Protein)  (3)(sensitiv or sensitivity and specificity or predictive or predictive value of tests or accuracy) |
| ***Search strategy for Scopus***  (1)"Liver Cirrhosis" OR "Hepatic Cirrhosis" OR "Cirrhosis, Hepatic" OR "Cirrhosis, Liver" OR "Fibrosis, Liver" OR "Liver Fibrosis"  (2)"Chitinase 3-like protein 1" OR "Chitinase 3 Like Protein 1" OR "Cartilage Glycoprotein 39" OR "Glycoprotein 39, Cartilage" OR "GP-39 Protein" OR "GP 39 Protein" OR "YLK-40 Protein" OR "YLK 40 Protein" OR "CHI3L1 Protein" OR "CGP-39 Protein" OR "CGP 39 Protein"  (3)"sensitiv" OR "sensitivity and specificity" OR "predictive" OR "predictive value of tests" OR "accuracy" |
| ***Search strategy for Cochrane Library***  (1)(Hepatic Cirrhosis):ab,ti,kw OR (Cirrhosis, Hepatic):ab,ti,kw OR (Cirrhosis, Liver):ab,ti,kw OR (Fibrosis, Liver):ab,ti,kw OR (Liver Fibrosis):ab,ti,kw  (2)(Chitinase 3 Like Protein 1):ab,ti,kw OR (Cartilage Glycoprotein 39):ab,ti,kw OR (Glycoprotein 39, Cartilage):ab,ti,kw OR (GP-39 Protein):ab,ti,kw OR (GP 39 Protein):ab,ti,kw OR (YLK-40 Protein):ab,ti,kw OR (YLK 40 Protein):ab,ti,kw OR (CHI3L1 Protein):ab,ti,kw OR (CGP-39 Protein):ab,ti,kw OR (CGP 39 Protein):ab,ti,kw  (3)(sensitiv):ab,ti,kw OR (sensitivity and specificity):ab,ti,kw OR (predictive):ab,ti,kw OR (predictive value of tests):ab,ti,kw OR (accuracy):ab,ti,kw |
| ***Search strategy for Sinomed***   ("诊断"[不加权:扩展]) AND ((CHI3L1) OR(壳酶蛋白) ("壳多糖酶3样蛋白1"[不加权:扩展])) AND ((肝硬化) OR ("肝纤维化"[不加权:扩展])) |
| ***Search strategy for the CNKI***  主题=肝硬化 or 题名 = 肝纤维化 and 主题 = 壳多糖酶3样蛋白1 or 题名 = 壳酶蛋白 or 主题 = CHI3L1 and 摘要 = 诊断 and (精确匹配) |
| ***Search strategy for the Chinese Medical Journal Database***  (((题名或关键词=肝硬化 OR 题名或关键词=肝纤维化) AND (题名或关键词=壳多糖酶3样蛋白1 OR 题名或关键词=壳酶蛋白 OR CHI3L1)) AND 文摘=诊断) |
| ***Search strategy for the Wanfang Database***  主题:(肝硬化+肝纤维化) and 主题:(壳多糖酶3样蛋白1+壳酶蛋白+CHI3L1) and 主题:(诊断) |
