# Supplementary material for: Diagnostic Value of Serum Chitinase-3-Like Protein 1 for Liver Fibrosis: A Meta-analysis
Source: Biomed Res Int. 2022 Mar 20;2022:3227957. doi: 10.1155/2022/3227957 (PMC8961437; doi:10.1155/2022/3227957)
Supplement: Supplementary 2 — Supplementary Table 2: characteristics of the included studies. [file 3227957.f2.docx]

Supplementary Table 2. Characteristics of the included studies

|  | Yang et al  (n=119) | Huang et al  (n=244) | Kumagai et al  (n=134) | Mehta et al  (n=74) | Mo et al  (n=460) | Pan  et al  (n=59) | Yu et al  (n=246) | Saitou et al  (n=109) | Tran et al  (n=146) | Wu et al  (n=134) | Xiao  et al  (n=172) |
| --- | --- | --- | --- | --- | --- | --- | --- | --- | --- | --- | --- |
| **Fibrosis stage ≥1** | | | | | | | | | | | |
| Cut-off | NA | NA | NA | NA | 81.6ng/mL | / | NA | NA | NA | NA | NA |
| AUROC |  |  |  |  | 0.83 | 0.79 |  |  |  |  |  |
| TP |  |  |  |  | 221 | 28 |  |  |  |  |  |
| FP |  |  |  |  | 4 | 3 |  |  |  |  |  |
| FN |  |  |  |  | 151 | 6 |  |  |  |  |  |
| TN |  |  |  |  | 84 | 22 |  |  |  |  |  |
| Sensitivity |  |  |  |  | 0.59 | 0.82 |  |  |  |  |  |
| Specificity |  |  |  |  | 0.95 | 0.88 |  |  |  |  |  |
| PPV |  |  |  |  | 0.98 | 0.85 |  |  |  |  |  |
| NPV |  |  |  |  | 0.36 | 0.90 |  |  |  |  |  |
| **Fibrosis stage ≥2** | | | | | | | | | | | |
| Cut-off | 32.2ng/mL | 72.03ng/mL | NA | 95.5ng/mL | 85.1ng/mL | NA | NA | 186.4ng/mL | NA | 75.95ng/mL | NA |
| AUROC | 0.83 | 0.94 |  | 0.66 | 0.85 |  |  | 0.80 |  | 0.73 |  |
| TP | 53 | 152 |  | 38 | 171 |  |  | 62 |  | 26 |  |
| FP | 16 | 10 |  | 15 | 36 |  |  | 6 |  | 18 |  |
| FN | 12 | 17 |  | 12 | 63 |  |  | 15 |  | 18 |  |
| TN | 91 | 65 |  | 9 | 190 |  |  | 26 |  | 72 |  |
| Sensitivity | 0.82 | 0.90 |  | 0.76 | 0.73 |  |  | 0.80 |  | 0.60 |  |
| Specificity | 0.85 | 0.87 |  | 0.38 | 0.84 |  |  | 0.81 |  | 0.76 |  |
| PPV | 0.77 | 0.94 |  | 0.72 | 0.83 |  |  | 0.80 |  | 0.59 |  |
| NPV | 0.88 | 0.79 |  | 0.43 | 0.75 |  |  | 0.79 |  | 0.80 |  |
| **Fibrosis stage ≥3** | | | | | | | | | | | |
| Cut-off | 33.3ng/mL | 78.48ng/mL | 165ng/mL | NA | 84.6ng/mL | NA | NA | NA | NA | NA | NA |
| AUROC | 0.92 | 0.96 | 0.76 |  | 0.85 |  |  |  |  |  |  |
| TP | 37 | 159 | 69 |  | 110 |  |  |  |  |  |  |
| FP | 12 | 9 | 8 |  | 64 |  |  |  |  |  |  |
| FN | 4 | 10 | 30 |  | 45 |  |  |  |  |  |  |
| TN | 119 | 66 | 27 |  | 251 |  |  |  |  |  |  |
| Sensitivity | 0.90 | 0.94 | 0.70 |  | 0.76 |  |  |  |  |  |  |
| Specificity | 0.91 | 0.88 | 0.77 |  | 0.8 |  |  |  |  |  |  |
| PPV | 0.76 | 0.95 | 0.68 |  | 0.63 |  |  |  |  |  |  |
| NPV | 0.97 | 0.87 | 0.78 |  | 0.85 |  |  |  |  |  |  |
| **Fibrosis stage 4** | | | | | | | | | | | |
| Cut-off | 33.3ng/mL | NA | NA | 141.75ng/mL | NA | NA | 92.25ng/mL | 284.8ng/mL | 330ug/mL | NA | / |
| AUROC | 0.89 |  |  | 0.75 |  |  | 0.93 | 0.81 | NA |  | 0.74 |
| TP | 15 |  |  | 21 |  |  | 91 | 24 | 66 |  | 75 |
| FP | 49 |  |  | 15 |  |  | 11 | 18 | 2 |  | 35 |
| FN | 0 |  |  | 7 |  |  | 21 | 6 | 63 |  | 15 |
| TN | 108 |  |  | 31 |  |  | 103 | 61 | 15 |  | 55 |
| Sensitivity | 1.00 |  |  | 0.75 |  |  | 0.81 | 0.80 | 0.51 |  | 0.83 |
| Specificity | 0.69 |  |  | 0.67 |  |  | 0.90 | 0.77 | 0.89 |  | 0.61 |
| PPV | 0.23 |  |  | 0.58 |  |  | 0.89 | 0.73 | 0.97 |  | 0.68 |
| NPV | 1.00 |  |  | 0.82 |  |  | 0.83 | 0.78 | 0.19 |  | 0.79 |
